# Attitudes and Beliefs towards Sport Specialization, College Scholarships, and Financial Investment among High School Baseball Parents

**DOI:** 10.3390/sports7120247

**Published:** 2019-12-10

**Authors:** Eric G. Post, Michael D. Rosenthal, Mitchell J. Rauh

**Affiliations:** School of Exercise and Nutritional Sciences, San Diego State University, San Diego, CA 92182, USA; mrosenthal@sdsu.edu (M.D.R.); mrauh@sdsu.edu (M.J.R.)

**Keywords:** youth sport, survey research, socioeconomic status, club sports

## Abstract

Adolescent athletes are increasingly encouraged to specialize in a single sport year-round in an effort to receive a college scholarship. For collegiate baseball, only 11.7 scholarships are available for a 35-player team. The beliefs of the parents of baseball athletes towards sport specialization are unknown, along with whether they have an accurate understanding of college baseball scholarship availability. The parents of high school baseball athletes were recruited to complete an anonymous questionnaire that consisted of (1) parent and child demographics, (2) child baseball participation information, and (3) parent attitudes and beliefs regarding sport specialization and college baseball scholarships. One hundred and fifty-five parents participated in the questionnaire (female: 52.9%, age: 49.4 ± 5.5 years old). The parents spent a median of 3000 USD [Interquartile Range (IQR): 1500–6000] on their child’s baseball participation. Most parents believed that specialization increased their child’s chances of getting better at baseball (N = 121, 79.6%). The parents underestimated the number of college baseball scholarships available per team (median [IQR]: 5 [0–5]), but 55 parents (35.9%) believed it was likely that their child would receive a college baseball scholarship. Despite having a realistic understanding of the limited college scholarships available, the parents were optimistic that their child would receive a baseball scholarship.

## 1. Introduction

With nearly half a million annual participants, baseball is the fourth most popular boy’s high school sport in the United States, behind only football, track and field, and basketball [1]. However, there has been concern regarding the increasing occurrence of serious upper extremity injuries in youth baseball, with a recent study reporting that 57% of all ulnar collateral ligament (UCL) reconstructions are now performed on adolescent pitchers [2]. In response to this alarming rise in youth athlete injury and surgery rates, Major League Baseball and USA Baseball developed the Pitch Smart initiative, which provides pitch volume and sport participation guidelines to parents, coaches, and youth athletes [3]. Several of these recommendations pertain to year-round intensive training in a single sport, also known as sport specialization, which has been increasingly identified as a risk factor for overuse injury in youth sports [4,5,6]. These recommendations include not pitching for 3–4 months, playing other sports during the course of the year, and avoiding playing baseball for multiple teams at the same time [3]. While youth baseball players have been found highly compliant with pitch and inning count guidelines, they are less compliant with guidelines regarding sport specialization and year-round play, which are less enforceable by high school leagues and may be determined more by parent and child attitudes, beliefs, and access [7].

Previous research has attempted to determine the factors that have led to the rise in early sport specialization in modern youth sports by examining the attitudes and beliefs of adolescent athletes and their parents [8,9]. While parents recognize specialization as a risk factor for injury, this awareness is potentially outweighed by the overwhelming belief of adolescent athletes that specialization is important for the optimal development of sport skills and, ultimately, making a college team [8,9]. Parent socioeconomic status (SES) has also been identified as a key factor in early sport specialization, with parents from higher SES backgrounds being more likely to have highly specialized children that participate in pay-to-play club teams [10]. Additionally, a sizable proportion of adolescent athletes report that they believe they will receive a college athletics scholarship [8]. These findings indicate that parents might also have unrealistic expectations regarding their child’s chances of receiving a college scholarship or have an inaccurate understanding of the actual availability of college athletic scholarships. For example, a National Collegiate Athletic Association (NCAA) Division I baseball program only has 11.7 scholarships available that it can distribute across a maximum of 27 players on a 35-player roster [11]. Furthermore, it is also unknown whether the parental attitudes and beliefs towards the potential benefits of sport specialization in adolescent baseball athletes are similar to those reported in prior studies of adolescent athletes from various sports [12]. The parents of baseball athletes in particular may hold different attitudes and beliefs as a result of the MLB Pitch Smart campaign, which has been actively discouraging specialization at all levels of youth baseball since the start of the dissemination campaign in 2014 [3].

Therefore, the primary purpose of this study was to describe the attitudes, beliefs, and perceived barriers of the parents of high school baseball players regarding sport specialization and college scholarships. A secondary purpose was to examine the potential differences in child baseball participation characteristics based on parent median household income (MHI). We hypothesized that (1) most parents would believe specialization increases the risk of a baseball-rated injury, (2) an even larger proportion of parents would believe specialization is beneficial for sport development and making a college team, (3) the parents would overestimate the availability of Division I baseball scholarships, and (4) most parents would believe that their child was likely to receive a college baseball scholarship. Finally, we hypothesized that parents in higher MHI categories would spend more money on their child’s baseball participation and would be more likely to have a child that is highly specialized in baseball, participating in a club team and participating in baseball year-round, compared to lower MHI parents.

## 2. Materials and Methods

### 2.1. Participants

The parents of high school baseball athletes from six high schools (median student enrollment: 2051 [1732.5–2373.8], median proportion of students eligible for free/reduced lunch: 26.8% [11.1–50.0%]) in San Diego county were recruited to complete an anonymous questionnaire during the 2019 spring high school baseball season. The parents were recruited during pre-season baseball participation meetings and had the option of completing either a paper-and-pencil or online version of the questionnaire (Qualtrics, Provo, UT, USA). The parents were eligible to participate in the study if they had a child that was a member of the high school’s varsity, junior varsity, or freshman baseball teams. This study was approved by the Institutional Review Board of San Diego State University. The parents provided oral consent prior to participation in the study due to the anonymous nature of the questionnaire.

### 2.2. Questionnaire

The parents completed an anonymous questionnaire that was developed for this study and adapted from previous research on the knowledge, attitudes, and beliefs regarding sport specialization among youth sport parents and coaches [9,13]. The questionnaire consisted of (1) parent and child demographics, including parent sex, age, race, educational attainment, residential zip code, child age, and school grade; (2) child baseball participation information, such as months per year and hours per week of baseball participation, sport specialization status, club baseball team participation, number of overnight trips for baseball participation in previous year, money spent on child’s baseball participation in previous year, and whether their child had sustained a baseball-related injury in the previous year; (3) parent attitudes and beliefs regarding sport specialization and college baseball scholarships; and (4) perceived barriers to baseball participation.

The questionnaire was developed using a panel of content-area experts using the Health Belief Model (HBM) as a framework. The HBM states that health behavior is determined by personal beliefs and perceptions regarding a disease or condition. The four constructs of the HBM are: (1) perceived seriousness of a condition, (2) perceived susceptibility to a condition, (3) perceived benefits of altering behavior, and (4) perceived barriers to altering behavior [14,15]. The questions regarding attitudes, beliefs, and perceived barriers regarding sport specialization and baseball participation were designed to address these constructs with sport specialization as the condition of interest.

The child sport specialization status (low, moderate, high) was determined using a validated 3-point specialization scale [4,5]. Months per year and hours per week of child baseball participation were used to classify children as participating more than 8 months per year (yes/no) and participating in more hours per week than their age (yes/no), based on previous sport volume recommendations. All of the financial variables were reported in U.S. dollars (USD). The residential zip code was used to estimate the median household income (MHI) using United States Census Bureau data. The sample was then stratified into MHI tertiles (low MHI, middle MHI, high MHI) for analysis.

### 2.3. Statistical Analysis

The data were summarized using means and standard deviations (SD), medians and interquartile ranges [IQR], and frequencies and proportions (%). The continuous variables were assessed for normality using the visual inspection of histograms and skewness/kurtosis values. The normally distributed variables were presented as means (SD), and the non-normal variables were presented as medians [IQR]. Chi-square tests were used to compare the frequency of child sport specialization status and sport participation characteristics by parent MHI tertile. The Kruskal–Wallis H-test was used to compare the amount of money spent on their child’s baseball participation in the previous year between parent MHI tertiles. Post-hoc pairwise comparisons between MHI tertile pairs were conducted using the Dunn test for multiple comparisons with a Holm–Bonferroni correction. An alpha level of 0.05 was set a priori to determine the statistical significance for all tests. All analyses were performed using R statistical software (R Foundation for Statistical Computing, Vienna, Austria).

## 3. Results

The parent and child descriptive data are provided in Table 1.

One hundred and fifty-five parents of high school baseball athletes participated (female: 52.9%, age: 49.4 ± 5.5 years old). Most parents reported a bachelor’s or higher level of education (N = 113, 73.8%) and reported their racial identity as white/Caucasian (N = 119, 78.8%). The median household income for the entire sample was 99,250 USD [IQR: 77,361–120,231]. The mean child age reported by the parents was 15.8 ± 1.2 years old. The child sport participation characteristics are presented in Table 2.

Approximately half of all of the children (N = 73, 47.7%) were classified as highly specialized in baseball by their parents. Over two-thirds of the parents reported that their child played on a club baseball team in addition to their high school team (N = 110, 71.0%), and nearly four in five parents reported that their child participated in baseball year-round (N = 119, 76.8%). The parents reported spending a median of 3000 USD [IQR: 1500–6000] on their child’s baseball participation in the previous year.

The parents’ attitudes and beliefs regarding sport specialization are presented in Table 3.

**Figure 1:**
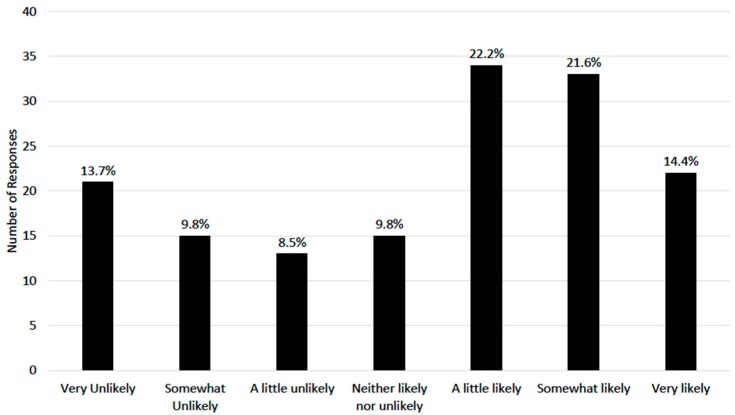
“How likely do you believe it is that your child will receive a college scholarship to play baseball?”.

Only 28.4% of the parents were “very” or “extremely” concerned about the risk of injury in youth sports. Most parents believed that specialization increased their child’s chances either “quite a bit” or “a great deal” for getting better at baseball (N = 121, 79.6%) and making a college team (N = 107, 70.0%). Fewer than half of all parents believed that specialization would increase the chances of their child getting injured either “quite a bit” or “a great deal” (N = 72, 47.1%). The parents underestimated the number of college baseball scholarships available per team (median estimate [IQR]: 5 [0–5], compared to actual value of 11.7 scholarships per team). However, 55 parents (36.0%) believed it was “somewhat” or “very likely” their child would receive a college baseball scholarship (Figure 1).

**Figure 2:**
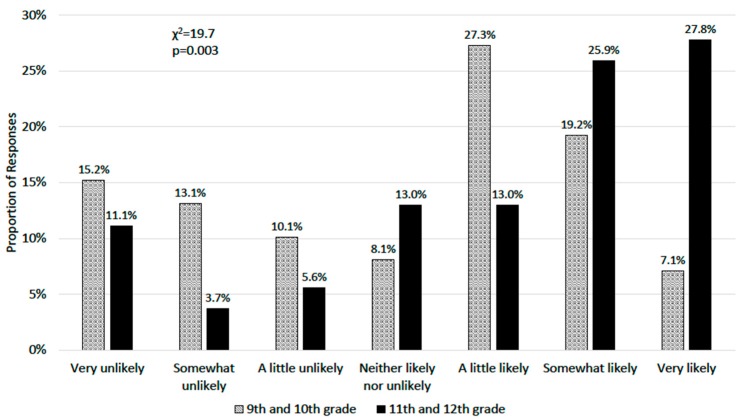
Comparison between the parents of underclassmen (9th or 10th grade) and upperclassmen (11th or 12th grade) in response to “How likely do you believe it is that your child will receive a college scholarship to play baseball?”.

The parents of upperclassmen (11th or 12th grade) were more likely to believe it was somewhat or very likely (53.7%) that their child would receive a college baseball scholarship compared to underclassmen (9th or 10th grade) parents (26.3%, *p* = 0.003) (Figure 2).

Perceived barriers to child baseball participation are presented in Table 4.

Overall, the travel requirements for tournaments and showcases (N = 31, 20.0%) and the time demands of non-sport activities (N = 24, 15.6%) were the potential barriers that the most parents indicated represented “quite a bit” or “a great deal”. However, for each potential reason more than 50% of all parents responded that it was “not at all” or just “a little” barrier.

The differences in child sport participation based on MHI tertile are presented in Table 5.

**Figure 3:**
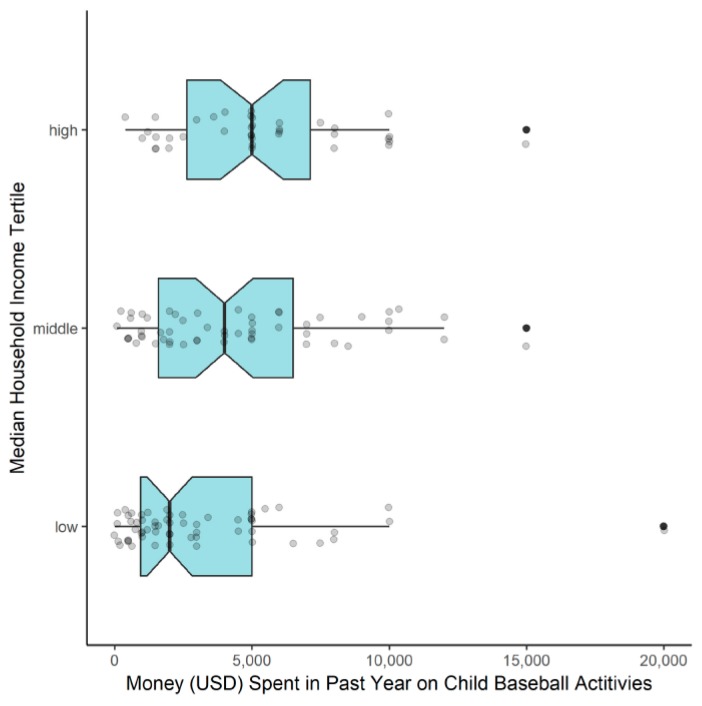
Comparison of money spent in past year on child’s baseball activities between parent MHI tertiles. Presented as a notched box plot, with box representing interquartile range (IQR), whiskers representing range of 1.5 times the IQR, line indicating median, notch displaying 95% confidence interval of the median, and individual data points for each parent (outliers in black).

High MHI parents were more likely to classify their child as highly specialized (65.8%) compared to middle MHI (51.9%) or low MHI (31.7%) parents (*p* = 0.02). Similarly, high MHI parents were more likely to report that their child participated in a club baseball team (82.1%) compared to middle MHI (76.4%) or low MHI (58.3%) parents (*p* = 0.02) and more likely to report that their child played baseball year-round (high MHI: 87.2%, middle MHI: 80.0%, low MHI: 66.7%, *p* = 0.047). When comparing the amount of money spent on their child’s baseball in the previous year, high MHI (5000 USD [2625–7125], *p* < 0.001) and middle MHI parents (4000 USD [1600–6500], *p* = 0.03) spent significantly more money compared to low MHI parents (2000 USD [950–5000]) (Figure 3).

## 4. Discussion

The most important finding of this study was that 70–80% of parents believed that specialization in baseball would improve their child’s baseball ability and chances of making a college team. Conversely, only 47% of parents believed that specialization would increase their child’s chances of sustaining an injury. These results are consistent with previous research in a larger sample of 1000 parents of adolescent athletes from various sports, which reported that only 43% of parents believed that specialization increased the chances of overuse injury [9]. Previously, sport specialization has been repeatedly identified as a risk factor for injury in various sports [4,5,6,16], although evidence for an association between specialization in baseball and overuse injury is mixed [17]. Our results indicate that many parents may not be aware of the research linking specialization with an increased risk of injury, or perhaps more likely, view the potential benefits of specialization as outweighing the potential injury consequences. The belief that specialization greatly improves sport ability appears to be widespread. Brooks et al. reported that most adolescent athletes view specialization as beneficial for improving sport performance (91%) or making a college team (67%), similar to the large majority of parents in our study who indicated similar beliefs [8]. Similarly, in a survey of 201 youth sport parents, Padaki et al. reported that 57% of parents hoped for their child to play either collegiately or professionally, and half of all parents reported that they had encouraged their child to specialize in a single sport [18]. To our knowledge, this is the first study to examine the perceived benefits of specialization among parents of adolescent baseball athletes.

Despite this widespread belief in the benefits of specialization for developing sport ability, previous evidence has suggested that specialization may not be necessary to advance to elite levels of sport [19,20,21,22]. In baseball specifically, a survey of 708 minor league professional baseball players reported that 64% of all players specialized in baseball after the age of 16, and that most players followed a pattern of sampling many sports in childhood before ultimately specializing in late adolescence [19]. This pattern of early sampling followed by eventual specialization is consistent with the Developmental Model of Sport Performance, which describes both early specialization and early sampling as pathways that can lead to elite performance [23,24]. Retrospective studies in NCAA Division I athletes [22], national team athletes in the United Kingdom [21], and elite Danish athletes [20] have all reported that specialization was not necessary for elite performance and instead most athletes followed the early sampling pathway. Additionally, the specialization patterns followed by professional baseball players also closely match the Long-Term Development Plan developed by USA Baseball, which also emphasizes the importance of fun and sport sampling early in an athlete’s development, followed by a gradual increase in sport-specific training across the course of adolescence [25]. The disconnect between the existing body of literature on sport development, specialization, and overuse injury with parent attitudes and beliefs is concerning, as parents are a primary decision-maker regarding a child’s sport participation [26].

To our knowledge, this is the first study to examine parental beliefs regarding the availability of college athletics scholarships and their child’s chances of receiving a college athletic scholarship. We hypothesized that parents would overestimate the availability of college baseball scholarships (i.e., believe that there are more than 11.7 scholarships available per team), but interestingly, the parents in this study actually underestimated the amount of scholarships available per team. This level of understanding of baseball scholarship availability may be partially due to the specifics of the sample recruited for this study (highly educated, affluent, large financial investment in child’s baseball). However, while the parents had an overly realistic understanding of college baseball scholarship availability, over a third of parents (36%) still believed that is was “somewhat” or “very” likely that their child would receive a college baseball scholarship. In fact, more than half of all parents (58.2%) believed it was at least “a little” likely or more that their child would receive a baseball scholarship. In contrast, previous data suggest that only between 2.2% and 5.7% of all high school baseball players will play college baseball, and even fewer will receive a scholarship to do so [11,27]. Our results suggest that even among parents that have a realistic understanding of the rarity of college athletic scholarships, a disconnect exists between that understanding and personal belief in their child’s likelihood of receiving a scholarship. We also found that the parents of upperclassman had a stronger belief in their child’s chances of receiving a college scholarship compared to parents of underclassman. This may reflect that their children were in fact in the process of being recruited by colleges or that these parents had a different perception of their child’s chances of playing college baseball as their child neared the end of high school. As this study’s design was cross-sectional in nature, prospective cohort studies are needed that can assess the parents’ beliefs about specialization and their child receiving a scholarship, and then track the youth baseball players until the start of their college years to observe who received and who did not receive a scholarship.

The parents reported higher rates of child specialization and club sport participation compared to previous research. Nearly half (47.7%) of the parents reported that their child was highly specialized and 71% reported that their child participated on a club baseball team in addition to their high school team. A previous study of 1544 high school athletes from various sports reported that 49% of all athletes also participate on a club team. Previous research estimating the prevalence of specialization has reported rates between 13% and 37%, with rates depending on a variety of factors including school size, athlete sex, age, sport, parent SES, and geographical region [10,19,28,29,30,31]. The specific sport (baseball) examined, the affluence of our sample, and the differences in geographical region from previous research (Southern California vs primarily Midwest United States) most likely contributed to the increased rates of specialization and club sport participation observed in this study.

As mentioned previously, parent SES has been identified in multiple studies as having a significant influence on sport specialization and youth sport participation characteristics [10,31]. Jayanthi et al. reported that high-SES youth athletes were more likely to be highly specialized and trained for more months per year and hours per week in their main sport [31]. In a study of 949 youth sport parents, the higher income parents were more likely to have a highly-specialized child, and, overall, the parents reported spending 1500 USD in the past year on their child’s sport participation [10]. We found that the high MHI parents were more likely than the low MHI parents to have a highly specialized child (65.8% vs. 31.7%), have a child participating on a club baseball team (82.1% vs. 58.3%), and have a child that plays baseball year-round (87.2% vs. 66.7%). Overall, the parents spent a median of 3000 USD [1500–6000 USD] on their child’s baseball participation, with the high MHI parents spending more than twice as much money on their child’s baseball participation compared to the low MHI parents. Our findings provide additional support to the existing literature that suggests that financial resources play a significant role in the ability to specialize and focus on a single sport year-round, which may limit the participation of individuals from families with more limited resources. For example, the greatest barrier to baseball participation reported by parents in our study were “travel requirements for tournaments and showcases”, which may be a particularly significant barrier for families with limited financial resources.

There are several important limitations to note in our study. First, our sample was primarily white/Caucasian (78.8%), highly educated (73.8% had bachelor’s degree or above), and affluent (MHI: 99,250 USD [77,631–120,231 USD]). Therefore, caution should be exercised in drawing conclusions regarding the broader population of youth sport parents, who may not hold similar attitudes or beliefs towards sport specialization. Clearly, further research is needed that includes larger, more diverse and representative youth sport populations. Furthermore, the participants were recruited from six high schools in just one region of the country (San Diego county), and thus our findings may not be generalizable to the parents of baseball athletes from different parts of the country. Finally, all of the data were self-reported by the parents, and so may have suffered from social desirability bias for certain responses regarding attitudes, beliefs, or behaviors. We estimated the MHI using residential zip code in an attempt to partially control the effect that the impact social desirability bias can have on personal income reporting, but this also presents its own limitations as we relied on an estimate of MHI for our analysis instead of actual parent-reported household income.

## 5. Conclusions

While almost 50% of high school baseball parents believed that specialization increases the risk of injury, an even higher proportion of parents believed that specialization greatly increases sport ability and the ability of their child to make a college baseball team. Despite having a realistic understanding of the limited number of college baseball scholarships available, parents were optimistic that their child would receive a baseball scholarship. The current year-round, highly specialized environment of youth sports in the United States may favor individuals from families with more financial resources.

## Figures and Tables

**Table 1:** Parent and child demographics.

Variable	N (%), Mean (SD), or Median [IQR]
**Parent Sex**	
Male	73 (47.1%)
Female	82 (52.9%)
**Parent Age**	49.4 (5.5%)
**Parent Race**	
Asian	8 (5.3%)
African American/Black	0 (0%)
American Indian/Alaskan Native	1 (0.7%)
Hispanic/Latino of any race	18 (11.9%)
Native Hawaiian/other Pacific Islander	0 (0%)
White/Caucasian	119 (78.8%)
Two or more races	5 (3.3%)
**Parent Education**	
Less than High School	0 (0%)
High school diploma or GED	26 (17.0%)
Associate or 2-year college degree	14 (9.2%)
Bachelor or 4-year college degree	66 (43.1%)
Graduate or professional degree	47 (30.7%)
**Parent Median Household Income (USD)**	99,250 [77,631–120,231]
**Parent Median Household Income Tertile**	
Low	60 (39.0%)
Middle	55 (35.7%)
High	39 (25.3%)
**Child Age**	15.8 (1.2)
**Child Grade**	
9th	58 (37.4%)
10th	41 (26.4%)
11th	26 (16.8%)
12th	30 (19.4%)

**Table 2:** Child sport participation characteristics. (N = 155).

Variable	N (%), Mean (SD), or Median [IQR]
**Total Number of Sports**	1.5 (0.6)
**Baseball Start Age**	5 [4–6]
**Years of Baseball Participation**	10.6 (2.0)
**Months/Year of Organized Baseball**	11 [9–12]
**Hours/Week of Organized Baseball**	12.3 (6.1)
**Hours/Week of Unorganized Baseball**	2 [1–5]
Specialization *	
Low	28 (18.3%)
Moderate	52 (34.0%)
High	73 (47.7%)
**Club Baseball Team Participation**	
Yes	110 (71.0%)
No	45 (29.0%)
**Participate in Baseball > 8 months per year**	
Yes	119 (76.8%)
No	36 (23.2%)
**Hours/Week of Organized Baseball > Child Age**	
Yes	34 (21.9%)
No	121 (78.1%)
**Number of Overnight Trips in Previous Year**	2 [0–5]
**Money Spent on Child’s Baseball in Previous Year (USD)**	3000 [1500–6000]
**Baseball-Related Injury in Previous Year**	
Yes	51 (32.9%)
No	104 (67.1%)

* 2 parents did not report child sport specialization data.

**Table 3:** Parent attitudes and beliefs regarding sport specialization.

Question	Response N (%)				
	Not at all	A little	Somewhat	Very	Extremely
**How concerned are you about the risk of injury in youth sports?**	18 (11.6%)	34 (21.9%)	59 (38.1%)	37 (23.9%)	7 (4.5%)
**How much does focusing on one sport and playing that sport all year increase your child’s chances of**	**Not at all**	**A little**	**Somewhat**	**Quite a bit**	**A great deal**
making a high school team?	7 (4.5%)	11 (7.1%)	35 (22.6%)	60 (38.7%)	42 (27.1%)
making a college team?	7 (4.5%)	12 (7.8%)	27 (17.7%)	46 (30.1%)	61 (39.9%)
getting injured?	6 (3.9%)	22 (14.4%)	53 (34.6%)	52 (34.0%)	20 (13.1%)
getting better at baseball?	1 (0.6%)	8 (5.3%)	22 (14.5%)	53 (34.9%)	68 (44.7%)
	**Not at all**	**A little**	**Somewhat**	**Quite a bit**	**A great deal**
**How much of a problem do you think early sport specialization is in youth sports?**	17 (11.0%)	17 (11.0%)	44 (28.4%)	42 (27.0%)	35 (22.6%)

**Table 4:** Perceived barriers to child baseball participation.

How Much do Each of the Following Reasons Limit Your Child’s Ability to Participate in Baseball?					
	Not at all	A little	Somewhat	Quite a Bit	A Great Deal
Cost (participation fees, equipment, coaching)	72 (46.4%)	43 (27.7%)	24 (15.5%)	10 (6.5%)	6 (3.9%)
Time demands of baseball	69 (44.8%)	38 (24.7%)	31 (20.1%)	10 (6.5%)	6 (3.9%)
Travel requirements for tournaments or showcases	55 (35.5%)	45 (29.0%)	24 (15.5%)	22 (14.2%)	9 (5.8%)
Ability to transport your child to local practices or events	91 (58.8%)	38 (24.5%)	20 (12.9%)	3 (1.9%)	3 (1.9%)
Time demands of other sports	98 (63.2%)	24 (15.5%)	22 (14.2%)	7 (4.5%)	4 (2.6%)
Time demands of non-sport activities (work, church, school, etc.)	37 (24.0%)	55 (35.7%)	38 (24.7%)	20 (13.0%)	4 (2.6%)
Family responsibilities	52 (33.8%)	53 (34.5%)	41 (26.6%)	7 (4.5%)	1 (0.6%)
Computer or TV-based entertainment	123 (79.4%)	18 (11.6%)	10 (6.5%)	1 (0.6%)	3 (1.9%)

**Table 5:** Differences in child sport participation based on MHI tertile.

Variable	Low MHI N (%)	Middle MHI N (%)	High MHI N (%)	X^2^	*p*
**Specialization**				11.6	0.02
Low	14 (23.3%)	9 (16.7%)	5 (13.2%)		
Moderate	27 (45.0%)	17 (31.5%)	8 (21.0%)		
High	19 (31.7%)	28 (51.8%)	25 (65.8%)		
**Club Baseball Participation**				7.7	0.02
Yes	35 (58.3%)	42 (76.4%)	32 (82.1%)		
No	25 (41.7%)	13 (23.6%)	7 (17.9%)		
**>8 months/year in baseball**				6.1	0.047
Yes	40 (66.7%)	44 (80.0%)	34 (87.2%)		
No	20 (33.3%)	11 (20.0%)	5 (12.8%)		
**Hours/week baseball > age**				1.8	0.42
Yes	16 (26.7%)	12 (21.8%)	6 (15.4%)		
No	44 (73.3%)	43 (78.2%)	33 (84.6%)		

## References

[1] High School Sports Participation Increases for 28th Straight Year Nears 8 Million Mark. https://www.nfhs.org/articles/high-school-sports-participation-increases-for-28th-straight-year-nears-8-million-mark/.

[2] Erickson B.J., Nwachukwu B.U., Rosas S., Schairer W.W., McCormick F.M., Bach B.R., Bush-Joseph C.A., Romeo A.A. (2015). Trends in medial ulnar collateral ligament reconstruction in the United States: A retrospective review of a large private-payer database from 2007 to 2011. Am. J. Sports Med..

[3] Pitch Smart. https://www.mlb.com/pitch-smart/risk-factors.

[4] Bell D.R., Post E.G., Biese K., Bay C., Valovich McLeod T. (2018). Sport Specialization and Risk of Overuse Injuries: A Systematic Review With Meta-analysis. Pediatrics.

[5] Jayanthi N.A., LaBella C.R., Fischer D., Pasulka J., Dugas L.R. (2015). Sports-specialized intensive training and the risk of injury in young athletes: A clinical case-control study. Am. J. Sports Med..

[6] Hall R., Barber Foss K., Hewett T.E., Myer G.D. (2015). Sport specialization’s association with an increased risk of developing anterior knee pain in adolescent female athletes. J. Sport Rehabil..

[7] Pytiak A.V., Stearns P., Bastrom T.P., Dwek J., Kruk P., Roocroft J.H., Pennock A.T. (2017). Are the current little league pitching guidelines adequate? A single-season prospective MRI study. Orthop. J. Sports Med..

[8] Brooks M.A., Post E.G., Trigsted S.M., Schaefer D.A., Wichman D.M., Watson A.M., McGuine T.A., Bell D.R. (2018). Knowledge, Attitudes, and Beliefs of Youth Club Athletes Toward Sport Specialization and Sport Participation. Orthop. J. Sports Med..

[9] Bell D.R., Post E.G., Trigsted S.M., Schaefer D.A., McGuine T.A., Brooks M.A. (2018). Parents’ Awareness and Perceptions of Sport Specialization and Injury Prevention Recommendations. Clin. J. Sport Med..

[10] Post E.G., Green N.E., Schaefer D.A., Trigsted S.M., Brooks M.A., Mcguine T.A., Watson A.M., Bell D.R. (2018). Socioeconomic status of parents with children participating on youth club sport teams. Phys. Ther. Sport.

[11] Baseball: Probability of Competing Beyond High School. http://www.ncaa.org/about/resources/research/baseball-probability-competing-beyond-high-school.

[12] Post E.G., Trigsted S.M., Schaefer D.A., Cadmus-Bertram L.A., Watson A.M., McGuine T.A., Brooks M.A., Bell D.R. (2018). Knowledge, Attitudes, and Beliefs of Youth Sports Coaches Regarding Sport Volume Recommendations and Sport Specialization. J. Strength Cond. Res..

[13] Glanz K., Rimer B., Lewis F. (2002). Health Behavior and Health Education.

[14] Rosenstock I.M., Strecher V.J., Becker M.H. (1988). Social learning theory and the Health Belief Model. Health Educ. Q..

[15] McGuine T.A., Post E.G., Hetzel S.J., Brooks M.A., Trigsted S., Bell D.R. (2017). A Prospective Study on the Effect of Sport Specialization on Lower Extremity Injury Rates in High School Athletes. Am. J. Sports Med..

[16] Norton R., Honstad C., Joshi R., Silvis M., Chinchilli V., Dhawan A. (2018). Risk Factors for Elbow and Shoulder Injuries in Adolescent Baseball Players: A Systematic Review. Am. J. Sports Med..

[17] Padaki A.S., Ahmad C.S., Hodgins J.L., Kovacevic D., Lynch T.S., Popkin C.A. (2017). Quantifying Parental Influence on Youth Athlete Specialization: A Survey of Athletes’ Parents. Orthop. J. Sports Med..

[18] Ginsburg R.D., Steven R., Danforth N., Ceranoglu T.A., Durant S.A., Kamin H., Babcock R., Robin L., Masek B. (2014). Patterns of Specialization in Professional Baseball Players. J. Clin. Sport Psychol..

[19] Moesch K., Elbe A.-M., Hauge M.-L.T., Wikman J.M. (2011). Late specialization: The key to success in centimeters, grams, or seconds (cgs) sports. Scand. J. Med. Sci. Sports.

[20] Bridge M.W., Toms M.R. (2013). The specialising or sampling debate: A retrospective analysis of adolescent sports participation in the UK. J. Sports Sci..

[21] Post E.G., Thein-Nissenbaum J.M., Stiffler M.R., Brooks M.A., Bell D.R., Sanfilippo J.L., Trigsted S.M., Heiderscheit B.C., McGuine T.A. (2016). High School Sport Specialization Patterns of Current Division I Athletes. Sports Health.

[22] Côté J., Lidor R., Hackfort D. (2009). To Sample or to Specialize? Seven Postulates About Youth Sport Activities that lead to continued participation and elite performance. Int. J. Sport Exerc. Psychol..

[23] Cote J., Vierimaa M. (2014). The developmental model of sport participation: 15 years after its first conceptualization. Sci. Sports.

[24] Long-Term Athlete Development Plan. http://www.sportdev.org/LTAD.

[25] Baxter-Jones A.D.G., Maffulli N. (2003). Parental influence on sport participation in elite young athletes. J. Sports Med. Phys. Fit..

[26] Malina R.M. (2010). Early sport specialization: Roots, effectiveness, risks. Curr. Sports Med. Rep..

[27] Post E.G., Bell D.R., Trigsted S.M., Pfaller A.Y., Hetzel S.J., Brooks M.A., McGuine T.A. (2017). Association of Competition Volume, Club Sports, and Sport Specialization With Sex and Lower Extremity Injury History in High School Athletes. Sports Health.

[28] Bell D.R., Post E.G., Trigsted S.M., Hetzel S., McGuine T.A., Brooks M.A. (2016). Prevalence of Sport Specialization in High School Athletics: A 1-Year Observational Study. Am. J. Sports Med..

[29] Bell D.R., Post E.G., Trigsted S.M., Schaefer D.A., McGuine T.A., Watson A.M., Brooks M.A. (2018). Sport Specialization Characteristics Between Rural and Suburban High School Athletes. Orthop. J. Sports Med..

[30] Jayanthi N.A., Holt D.B., LaBella C.R., Dugas L.R. (2018). Socioeconomic Factors for Sports Specialization and Injury in Youth Athletes. Sports Health.

[31] Post E.G., Trigsted S.M., Riekena J.W., Hetzel S., McGuine T.A., Brooks M.A., Bell D.R. (2017). The Association of Sport Specialization and Training Volume With Injury History in Youth Athletes. Am. J. Sports Med..

